# Influence of Inner Gas Curing Technique on the Development of Thermoplastic Nanocomposite Reinforcement

**DOI:** 10.3390/ma16227179

**Published:** 2023-11-15

**Authors:** Husam Saber Totah, Iqbal Ahmed Moujdin, Hani Abdulelah Abulkhair, Muhammad Albeirutty

**Affiliations:** 1 Department of Mechanical Engineering, King Abdulaziz University, P.O. Box 80200, Jeddah 21589, Saudi Arabia; 2 Center of Excellence in Desalination Technology, King Abdulaziz University, P.O. Box 80200, Jeddah 21589, Saudi Arabia

**Keywords:** curing, reinforced composite, shrinkage, tensile strength

## Abstract

In this work, a comprehensive shrinkage and tensile strength characterization of unsaturated polyester (UPE-8340) and vinyl ester (VE-922) epoxy matrices and composites reinforced with multiwall carbon nanotubes (MWCNTs) was conducted. The aspect ratio of UPE and VE with methyl ethyl ketone peroxide (MEKP) was kept at 1:16.6; however, the weight of the MWCNTs was varied from 0.03 to 0.3 gm for the doping of the reinforced nanocomposites. Using a dumbbell-shaped mold, samples of the epoxy matrix without MWCNTs and with reinforced UPE/MWCNT and VE/MWCNT nanocomposites were made. The samples were then cured in a typical ambient chamber with air and an inner gas (carbon dioxide). The effect of the MWCNTs on UPE- and VE-reinforced composites was studied by observing the curing kinetics, shrinkage, and tensile properties, as well as the surface free energy of each reinforced sample in confined saline water. The CO_2_ curing results reveal that the absence of O_2_ shows a significantly lower shrinkage rate and higher tensile strength and flexural modulus of UPE- and VE-reinforced nanocomposite samples compared with air-cured reinforced nanocomposites. The construction that was air- and CO_2_-cured produced results in the shape of a dumbbell, and a flawless surface was seen. The results also show that smaller quantities of MWCNTs made the UPET- and VE-reinforced nanocomposites more stable when they were absorbed and adsorbed in concentrated salt water. Perhaps, compared to air-cured nanocomposites, CO_2_-cured UPE and VE nanocomposites were better at reducing shrinkage, having important mechanical properties, absorbing water, and being resistant to seawater.

## 1. Introduction

Unsaturated polyester (UPE) resins and vinyl ester (VE)-reinforced composites are frequently used in the desalination sector due to their low cost, adequate mechanical capabilities, and great resistance to water, oils, and other chemicals. VE resins may be significantly more resistant to alkalis, hypochlorite, peroxides, acids, and solvents than UPE [1]. Additionally, VE resins offer better elongation to failure and corrosion resistance than UPE, allowing for additional load to be applied to the reinforcement. In most cases, the UPE and VE resins are dissolved in styrene and employed as the matrix for reinforced composites. The performance of reinforced composite finishing is significantly influenced by the matrix interface between glass, carbon, and jute fibers [2,3]. However, because of the physical and chemical properties of the fibers, a weak interlock interphase develops, which leads to poor tensile properties in reinforced composites [4,5,6,7,8,9].

Single-wall and multiwall carbon nanotubes (SWCNTs and MWCNTs) could be used in epoxy-reinforced composites because of their low density, large interfacial area, strong mechanical properties, and stability at high temperatures [10,11]. As a result, researchers have been encouraged to assess a wide range of possible uses [11,12,13,14,15]. For over 10 years, it has been possible to make SWCNT and MWCNT matrix arrays (mate fiber) [16,17,18,19,20]. A great deal of research has focused on enhancing the stiffness and strength of epoxies or pure resins that contain discrete CNTs [21]. In order to better understand these composites’ enhanced capabilities, various studies have focused on the production of polymer- and CNT-reinforced composites. The CNT-based reinforced composites may have fallen below expectations. 

Theoretically, uniform dispersion in a polymer matrix is still a major difficulty due to the low molecular weight and density of CNTs. The most important problem with CNTs is agglomeration, which is caused by the van der Waals attraction between epoxy resins and CNTs. Because of this, many studies have found a number of methods to fix the problem of CNTs being spread out in a polymer matrix. These dispersion methods, such as chemical functionalization, in situ polymerizations, and ultimate physical blending, all have shortcomings. Another significant issue is the physiosorption behavior of CNTs, either functionalized or nonfunctionalized with the polymer matrix, which determines the effectiveness of load transmission and energy dissipation (internal, bulk flow kinetic, or system potential) under loading. The characteristics of CNTs mean that ensuring a uniform dispersion in a polymer matrix is a very difficult task, and chemical methods for CNT dispersion require considerable time and effort. 

In various polymer [22,23], metallic [24,25], and ceramic [26,27] matrices, a number of studies have used CNTs as the only reinforcement. It is incredibly challenging to distribute them evenly throughout the matrix. However, CNTs can be well dispersed in epoxy resins with the help of gentle stirring and sonication to make a useful nanocomposite matrix. Zhang et al. [28] used ultrasonic vibration to achieve the homogeneous dispersion of MWCNTs throughout a cycloaliphatic epoxy matrix. Their results revealed a significant increase in the tensile strength of the nanocomposite from 31.9 to 55.9 MPa, by adding only 0.05 weight percent of MWCNTs. Moreover, the issue of uniform dispersion has led to the development of a variety of innovative methods for the dispersion and superior arrangement of CNTs in a matrix [22,23,24,25,26,27]. Transmission electron micrographs showed that MWCNTs in an epoxy resin had a superior dispersion and distribution after plasma treatment [26]. MWCNTs that underwent plasma treatment demonstrated a more significant resistance to the spread of epoxy resin cracks [26]. The direct growth of CNTs on carbon fibers caused the absorption of CNTs into an epoxy matrix from coated carbon fibers. The primary reinforcement is provided by CNTs grown on carbon fibers, while CNTs grown on fibers serve as a secondary reinforcement [24].

Affordable solutions and effective production methods have been sought to improve the tensile strength, fracture resistance, and chemical tolerance properties of reinforced composites. A more practical use of composites has recently been prompted by non-autoclave-based composite fabrication technologies with competitive prices and excellent performance. Composites are relatively cost-effective and offer the ability to customize complicated structures with unique fiber-volume fractions. As a result, resin transfer molding has become increasingly important in the production of articles required to prevent oxidation and hydrolysis damage during reinforced composite tailoring and curing.

Resin transfer molding (RTM) techniques may use two-sided molds, which have expensive and inefficient tooling designed specifically for making large article components [21]. However, the vacuum-assisted resin transfer molding (VARTM) method can help reduce this expensive fabrication cost. Technically speaking, VARTM is a modification technique for the RTM process that pulls a resin or resin mixture through preforms to saturate them. The VARTM technique reduces overall reinforced composite costs by utilizing only a one-sided mold tool, a vacuum bag as the counter-mold, and preformed rather than pre-impregnated materials. Work has been carried out to increase the interlaminar strength and fracture toughness of laminated composites. This work has been paired with current trends and research focuses on CNT nanotechnology [21,25,26,27,28,29].

On the other hand, the polymerization of polycarbonate [30], polymethyl methacrylate [31], polystyrene [32], and polyamide [33] in the presence of CO_2_ has received a lot of attention. A few recent studies have reported the curing reaction among epoxy resins and precursors under high pressure (0.1 MPa to 8 MPa) of CO_2_ [34,35,36]. However, no research has been conducted on the curing kinetics of UPE/MWCNTs and VE/MWCNTs matrices in the presence of CO_2_ at normal pressure and temperature. Therefore, the main purpose of this study is to quantify and correlate the effects of various quantities of MWCNTs on the curing kinetics and shrinkage analysis of UPE and VE nanocomposite matrices, including the comprehensive properties of the tensile strength of air- and CO_2_-cured UPE and VE nanocomposites at presence. This was performed using experimental and empirical curve-fitting methodologies. Additionally, the impact of MWCNTs on the mechanical properties of reinforced nanocomposites and the surface characteristics of reinforced nanocomposites were studied.

## 2. Experiment

### 2.1. Materials

The UPE and VE thermosets used in this study consisted of five parts UPE and VE and 0.3 parts hardener (methyl ethyl ketone peroxide, or MEKP). The Siropol 8340 unsaturated polyester resin (UPE), Hetron 922 vinyl ester resin (VE), and MEKP were kindly supplied by Saudi Industrial Resins Limited, Jeddah, Saudi Arabia. The hydroxylated functionalized multi-wall carbon nanotubes (MWCNTs) with >96% purity and 8–18 nm diameters were purchased from Nanografi Nano Technology (Jena, Thuringen, Germany) and used in different quantities of 0.03–0.3 wt.% for reinforced composites. Pure water was produced in the lab using an RO Lab system (Purelab Option SR-7, Elga Veolia, Lane End, High Wycombe, UK). Seawater brine (57,000 mg/L) was obtained from the Water and Environmental Services Company (WESCO, Jeddah, Saudi Arabia) and Reverse Osmosis (SWRO) desalination plant located on the Red Sea coast in Jeddah, Saudi Arabia. 

#### Preparation of Dumbbell-Shaped Reinforced Nanocomposite

**Figure 1:**
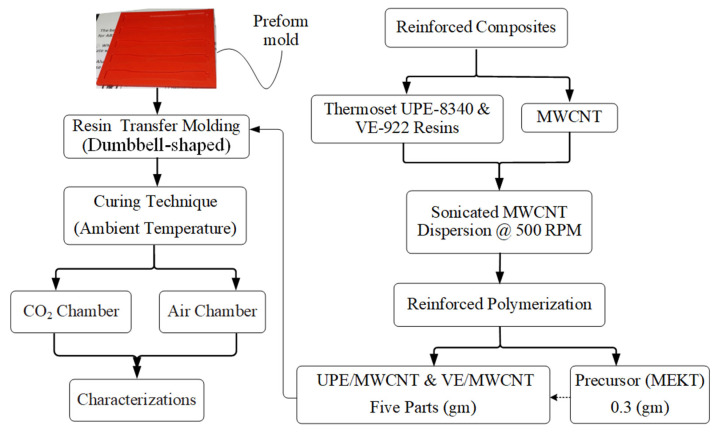
Schematic illustrations of the preparation protocol method for reinforced nanocomposites.

A dumbbell-shaped mold was designed according to ASTM D638–03 and tailored using 3D printers. The preparation method for the standard (Std) reinforced composite samples (without MWCNTs) and the dumbbell-shaped preform mold that was printed with a 3D printer has been explained in detail elsewhere [29]. Figure 1 shows a detailed protocol for the UPE- and VE-reinforced nanocomposite fabrication. Table 1 summarizes the composition of VE/MWCNT and UPE/MWCNT nanocomposites. Before being used to make the dumbbell-shaped reinforced nanocomposite samples, each thermoset (UPE and VE) resin was degassed for at least an hour in a vacuum oven at a vacuum pressure of 2 bar. After degassing and drying the thermoset resins and MWCNTs, the selected quantity of MWCNTs was loaded into each thermoset resin using a disposable polypropylene (PP) bottle.

A mechanical stirrer with a spring coil was used to mix the thermoset resin and MWCNTs at about 2000 rpm until the MWCNTs were completely mixed in. Each thermoset/MWCNT mixture in a PP bottle was sealed and placed in a Branson ultrasonic bath at 50 °C for at least 1 h. Before transferring the nanocomposite mixture into the dumbbell-shaped mold, the chosen amount of precursor (MEKP) was poured into each nanocomposite mixture and then mixed with a high-shear mechanical coil mixer set to 500 rpm for at least 10 min. Later, the sample was placed in a Branson ultrasonic bath at a higher sonication intensity (40 kHz) for at least 5 min to ensure no air bubbles remained inside the composite resin solution, and the bath temperature was kept at 10 °C ± 1 °C. The bench-scale experimental setup for the air- and CO_2_-cured resin transfer molding system is comprehensively described elsewhere [29].

## 3. Characterizations

### Shrinkage Analysis

The best composition of 5 parts of resin and 0.3 parts of MEKP was selected to fabricate the reinforced nanocomposite, and the shrinkage analysis was then carried out. The total shrinkage at ambient temperature (25 °C) of each cured, molded dumbbell-shaped specimen with and without CNTs was measured using a digital vernier caliper and evaluated using the following equation [29]:

(1) $$Total\;Shrinkage\;\left(\%\right)=\frac{\sum_{T=25}^{n_{1},n_{2},n_{3}}D_{m}-\sum_{T=25}^{n_{1},n_{2},n_{3}}D_{S}\;}{\sum_{T=25}^{n_{1},n_{2},n_{3}}D_{m}\;}\times 100$$

where *n*_1_, *n*_2_, and *n*_3_ are the width, length, and thickness of the mold and specimen, respectively; *T* is temperature (°C); *D_m_* is the 3D dumbbell-shaped mold size; and *D_s_* is the cured dumbbell-shaped specimen.

## 4. Tensile Strength

A 100 N load cell on an Instron 4411 Universal Tester was used to test the mechanical properties of reinforced composites in the shape of dumbbells that had been cured with air and CO_2_. All tests were carried out at room temperature with a crosshead speed of 50 mm/min. Three test specimens were used for each reinforced composite in line with BS EN ISO 527-2:1996. The mechanical properties were characterized using ASTM D790, which yielded values for the elastic modulus, flexural stress, and flexural strain. The ratio of stress to strain in the linear elastic area is typically known as the modulus of elasticity, also known as Young’s modulus. It is a measure of how stiff the material is and can be determined with the following equation:

(2) $$E=\frac{∆\sigma }{∆\varepsilon }$$

where *σ* is the stress and *ε* is strain.

## 5. Flexural Strength Test

The researchers investigated transverse rupture strength and flexural testing to learn more about the mechanical properties of the polymer matrix and reinforced nanocomposite matrix [37]. Each item was put through a flexural strength test in accordance with ASTM D 790 standards. This test is typically used to determine the maximum stress and strain caused by the addition of an external load. The specimens were tested on an Instron 4411 Universal Tester at 0.10 mm/min constant strain rate and 20 mm/min speed. The force accuracy was 0.5% of the applied load, and the speed resolution was 0.001 mm/min. All the samples were tested at room temperature, and every time, five specimens were tested for each composite to obtain average values. The dimensions of the samples were taken as a total length of 100 mm, 80 mm span length, 20 mm width, and 5 mm thickness. Each sample was set aside for three-point bending tests, and the strength of each polymer matrix and nanocomposite was calculated using the following relationship [37,38]:

(3) $$\sigma_{f}=\frac{3NL}{2wt^{2}}\;$$

where $\sigma_{f}$ is the flexural strength in MPa, *N* is maximum load, *L* is the length, *w* is width, and *t* is the thickness of dumbbell-shaped sample.

## 6. Flexural Modulus Test

The flexural modulus of the polymer composite matrix is distinct from the ability of that composite to deform. Technically, it is calculated from the slope of the stress and displacement curve. It is also called the tangent modulus or the modulus of elasticity. The following is usually used to obtain the flexural modulus [37,38]:

(4) $$E_{f}=\frac{L^{3}s}{4wt^{3}}$$

where $E_{f}$ is the flexural modulus, *s* is slope of the stress–strain curve, *L* is the length, *w* is width, and *t* is the thickness of dumbbell-shaped sample.

## 7. Interfacial Adhesion

With a modified version of the Kelly–Tyson model, interfacial shear stress can be used to ascertain how a MWCN spreads out in UPE and VE resins and how that affects how well nanotubes adhere to the resin.

(5) $$\tau_{NT}=\frac{\sigma_{CNTs}}{2\left(\frac{L_{c}}{D}\right)}\;\left(1-\frac{d_{CNTs}^{2}}{D_{CNTs}^{2}}\right)$$

The work involving pulling MWCNTs from a nanocomposite matrix is related to the interfacial shear stress by:

(6) $$W=\pi r\;\tau_{CNTs}L^{2}$$

where $\tau_{NT}$ is the failure stress, and *L_c_*, *d*, and *D*, are the critical length and inner and outer diameter of the CNTs, respectively. Table 2 shows the dimension descriptions of the MWCNTs used in this study.

## 8. Surface Characterization

### 8.1. Hygroscopicity and Wettability Analysis

This study primarily developed composite materials for tubes and fittings used in the desalination industry’s water transmission lines. The purpose of this test was to determine how much water the composite absorbs when exposed to seawater and fluxed water. To evaluate the absorption rate, each composite sample was individually placed in a closed test tube full of seawater and distilled water. The dimensions of each sample were chosen randomly. Each sample was dried in an oven for 12 h at 60 °C before being immersed in water. After drying, each sample was immersed in both water and air for 48 h at 25 ± 1 °C. The samples were examined in accordance with ASTM D-570. The water absorption of composite samples was calculated using the equation below.

(7) $$Absorp\;or\;Absorb_{water}\;\left(\%\right)=\frac{W_{w}-W_{d}}{W_{d}}\times 100$$

where *W_d_* is dry sample weight (g), *W_w_* is wet sample weight (g), *ρ_w_* is density of pure water and seawater (g/cm^3^), δ is thickness of the polymer matrix in a wet state (cm), and A is area of the polymer matrix in a wet state (cm^2^).

### 8.2. Contact Angle

An Attension Theta tensiometer was used to measure the contact angle of each polymer matrix using the Sessile drop method. Through a very fine capillary, 4 µL of deionized water droplets was applied to the polymer matrix surface, and the contact angle was determined dynamically using One Attension image analysis software v.4.1. 

### 8.3. Surface Free Energy Polymer Matrix

Apparent surface free energy $\epsilon_{s}$ of each specimen was calculated from the contact angle hysteresis (CAH) agreeing with the model proposed by Chibowski and Perea-Carpio [39], Chibowski et al. [40], and Chibowski [41]. The CAH approach relates $\epsilon_{s}$ to the surface tension of the probe liquid $\epsilon_{l}$ and CAH, which is defined as the difference between the advancing $\theta_{a}$ and receding $\theta_{r}$ contact angles

(8) $$\epsilon_{s}=\frac{\gamma_{l}{\left(1+\cos\theta_{a}\right)}^{2}}{2+\cos\theta_{a}+\cos\theta_{r}}$$

where $\epsilon_{s}$ is the apparent surface free energy, *l* is the water surface tension, and $\theta_{a}$ and $\theta_{r}$ are the advancing and receding contact angles of water.

## 9. Results and Discussion 

### 9.1. Dispersion of MWCNTs in Cured Nanocomposites

**Figure 2:**
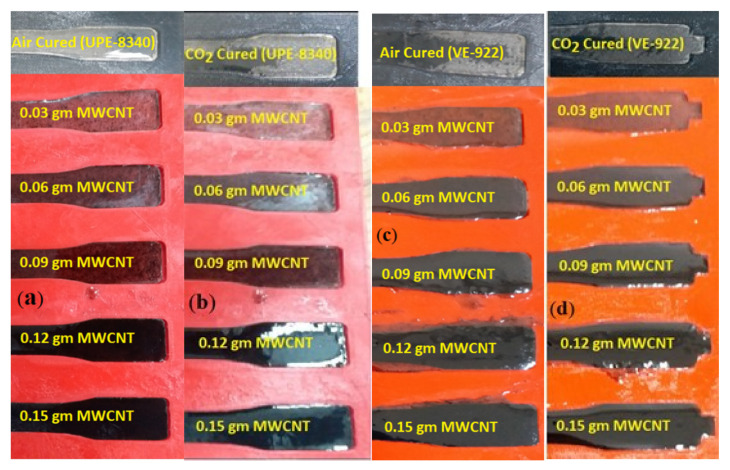
The shrinkage differences (air) between the 3D-printed ABS mold and epoxy specimen using various CNTs. (**a**)air-cured UPE nanocomposites; (**b**) CO_2_-cured UPE nanocomposites; (**c**) air-cured VE nanocomposites; (**d**) CO_2_-cured VE nanocomposites.

Based on the lowest shrinkage of air- and CO_2_-cured UPE-8340 and VE-922 matrices, the best ratio of resin (5 mL) to hardener (0.3 mL) was selected for the reinforced nanocomposite dumbbell-shaped structures [29]. At first, we attempted to disperse a higher concentration of MWCNTs (1 wt.%). In resins, perhaps because of the higher surface of MWCNTs, we faced severe issues of dispersion; consequently, we selected the lowest quantity of MWCNTs in both resins. Figure 2 shows that both polymer-reinforced nanocomposite samples (air- and CO_2_-cured) with a concentration of 0.03 wt% of MWCNTs had visible nanotube dispersions and kept their optical clarity with only a slight change in color. It can be clearly seen that the lowest quantity (0.03 to 0.09 gm) of MWCNTs in both the epoxy matrix and the polymer revealed itself to be effectively fused and aligned physically. These findings show that a high-shear mechanical coil mixer, followed by sonication at a longer duration, results in significant dispersions and alignment of MWCNTs in both resins. Even the higher quantities of MWCNTs (0.15 g) revealed significant dispersion, and it can be seen that the entire surface of the MWCNTs is covered by a polymer layer in both curing techniques. Gupta et al. [27] also used a higher-shear mechanical stirrer and a sonication technique to disperse the MWCNTs in Epon 862 epoxy resin. They reported that planetary shear mixing improved nanotube dispersion in epoxy. Moreover, to characterize the dispersion of MWCNTs in cured composite samples, they used three complementary techniques: optical microscopy, Raman spectroscopy, and scanning electron microscopy. For their cured sample, the optical micrographs and Raman images showed reduced agglomeration and a homogeneous distribution of MWCNTs in the epoxy matrix. Bower et al. [42] also reported that the lower quantity of MWCNTs can produce a strong bonding attachment with the polymer matrix. They validated their reinforced nanocomposite interpretation using transmission electron microscopy (TEM).

### 9.2. Shrinkage Analysis

In our earlier work [29], we thoroughly examined how various MEKP concentrations affected the kinetics of the curing process and the shrinkage characteristics of UPE-8340- and VE-922-reinforced matrices that were air- and CO_2_-cured. In this study, as shown in Table 1, we chose the best ratios of UPE and VE to MEKP to make reinforced nanocomposites. It has been demonstrated that by taking total shrinkage into account, a set of 3D-printed ABS plastic dumbbell molds can be accurately modeled. Dow Corning high-vacuum grease was used to lubricate the dumbbell-shaped ABS mold before each epoxy mixture was introduced. We showed that the warpage of a dumbbell-shaped specimen can be precisely replicated by considering total shrinkage (cure shrinkage plus thermal shrinkage). We examined the method of curing shrinkage to minimize it. A decrease in free volume is considered to correlate closely with cure shrinkage. 

Figure 2 shows the pinched dumbbell-shaped samples’ correlation between cure shrinkage and free volume for various combinations of air-cured and CO_2_-cured reinforced nanocomposites. The correlation between cure shrinkage and epoxy resin free volume supports the aforementioned hypothesis. Since the curing process is described in the section on curing and shrinkage at a temperature of 26 ± 1 °C (room temperature), the overall molded (lean epoxy matrix and nanocomposite matrix) data correspond to tests performed at this temperature. In Figure 2, it is observed that there is no physical or chemical effect on the 3D ABS mold. Consequently, before we transferred the nanocomposite dope into the mold, we covered it with a barrier of high-temperature silicon grease. This is likely why the nanocomposite sample pulled itself from the mold. The residual stresses within the resin develop as it cures, specifically in a greased, layered mold in that constrained environment within the spaces of the dumbbell shape. These stresses may exceed the inherent depth of both epoxy matrices, which vary in their degree of transformation. Moreover, the grease layer provides a stress-free ground for the epoxy resins during the curing process. 

Prior to selecting the best ratio of resin and MEKP, one must establish the approach to the development of a reinforced nanocomposite with low cure shrinkage. We followed a parameter that correlates closely with the cure shrinkage of both resins. We measured a variable that closely matched the shrinkage of both nanocomposites during curing, such as the displacement of each sample from the mold. Because the intermolecular distance is directly related to the quantity of MWCNTs used in both epoxy matrices, we hypothesized that cure shrinkage indicates a close correlation with the interaction of MWCNTs in each epoxy group. However, compared to UPE nanocomposites, the VE/MWCNTs show a significantly lower shrinkage rate. Typically, UPE resins have a higher viscosity than VE resins because of the presence of ethylene glycol and dibasic acid groups in UPE. Due to the hygroscopic properties of MWCNTs, the glycol molecules are most likely absorbed in MWCNTs, resulting in a more viscous UPE. The higher content of MWCNTs shows a lower shrinkage rate compared to lean epoxy resins. However, when compared to air-cured samples, CO_2_-cured samples exhibit less curing shrinkage displacement in the ABS mold. This is likely due to the absence of O_2_ and air contaminants present in air-cured samples. 

## 10. Influence of MWCNTs on Air and CO_2_-Cured Shrinkage of Nanocomposites

**Figure 3:**
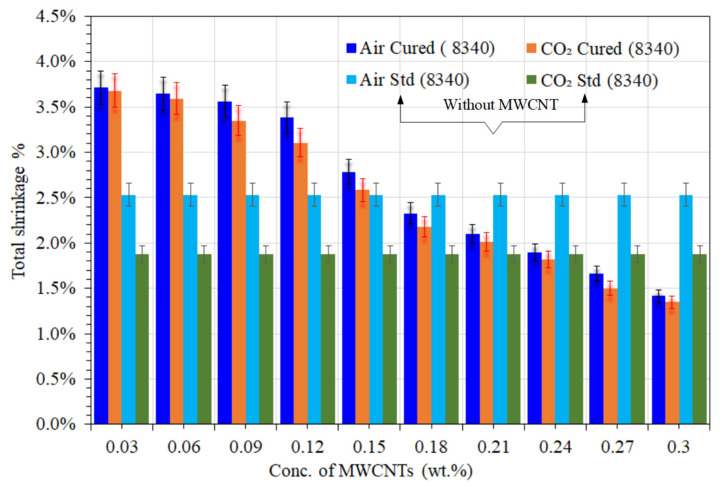
The shrinkage differences between air-cured and CO_2_-cured UPE-8340.

The total shrinkage of UPE-8340-reinforced nanocomposites utilizing both curing methods is shown in Figure 3. The nanocomposite matrices on the surface of the flat plate component of the mold cure more quickly than those in the spine, according to the shrinkage data from both curing processes, which is in line with the overall results. The volumetric shrinkage of UPE-8340 nanocomposites exhibits a linearly dependent profile with regard to low to high concentrations of MWCNTs in both curing techniques. CO_2_-cured samples, on the other hand, exhibit lower shrinkage kinetics than air-cured samples. This is most likely due to a lack of air-based gas scavengers and humidity. Lyu et al. [35] also reported that a minor amount of dissolved CO_2_ probably somewhat improves the curing reaction. 

Nevertheless, the lowest quantities of MWCNT-reinforced nanocomposites (0.03 to 0.15 ratio) showed a higher total shrinkage rate than conventional UPE-reinforced composites. This is most likely due to the presence of phthalic acid groups in UPE. The smallest quantity of CNTs may swell well with acidic media; thus, the interaction of carboxylic acid with MWCNTs may somewhat lead to functionalization, and thus the UPE chain becomes compact during polymerization [43]. According to Lee et al. [44], a mildly acidic nature encourages the fragmentation of smaller quantities of nanotubes to promote dispersion, and its viscous nature aids in inhibiting CNT reaggregation after dispersion in a PET matrix. However, when the air-curing method was used, the UPE-8340 nanocomposite matrices with a higher concentration of MWCNTs (0.18 to 0.3 wt.%) shrank slightly more. The same trends of shrinkage difference were also seen when a CO_2_ chamber was used to cure the material, but probably more so than with air-cured MWCNT matrices, CO_2_-cured nanocomposite showed a generally low shrinkage rate. Nevertheless, Figure 3 shows clear differences between the two curing methods in the shrinkage rate of UPE-8340 nanocomposite samples as a function of MWCNT concentration. 

However, unlike UPE-8340 nanocomposites, the overall VE-922 nanocomposite matrices show a lower shrinkage rate (see Figure 2), even with the lean reinforced composites in both curing techniques. A higher concentration of MWCNTs may result in a significant reduction in the shrinkage rate. The higher quantity of MWCNTs participating as filler can be interpreted here, and a number of studies [45,46,47] have reported that the filler can reduce the shrinkage rate of UPE- and VE-reinforced composites. 

**Figure 4:**
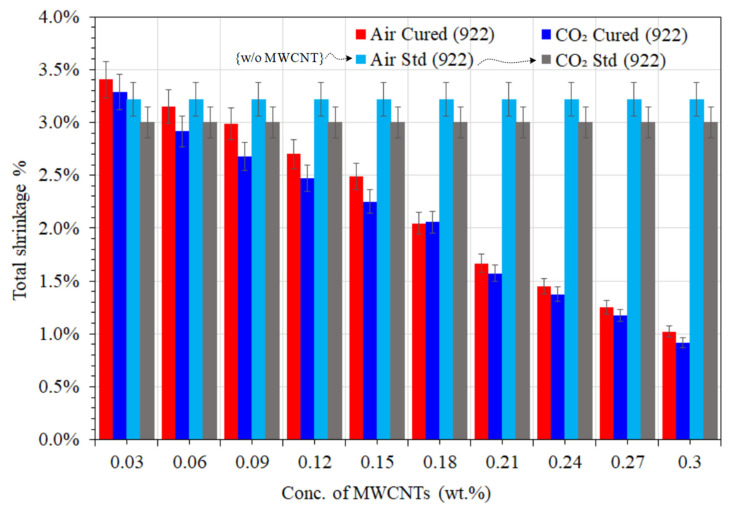
The shrinkage differences between air-cured and CO_2_-cured VPE-922.

Compared to a lean epoxy matrix (without MWCNTs), the samples containing MWCNTs in both reinforced nanocomposites showed faster curing and somewhat lower shrinkage behavior in air and CO_2_ curing, which was about 0.8% to 3.48%. The faster curing showed that MWCNTs are good thermal conductors, and the much smaller shrinkage showed that MWCNTs are aligned in the linear structural chain of all thermoset nanocomposite specimens. Figure 3 and Figure 4 show that the higher the amount of the MWCNT nanocomposite, the lower the shrinkage compared to the lean epoxy matrix. Figure 3 and Figure 4 also show that the CO_2_-cured nanocomposite sample showed significant development and revealed less shrinkage behavior than the air-cured specimen. Moreover, a higher quantity of MWCNTs was found to be more effective in terms of the lowest shrinkage, and Figure 2 shows good agreement, validating these results. It might be that the CNTs in the polymer matrix encourage the chemical reaction between resins and precursors, and the MWCNTs probably also facilitate the reduction in the carbon chain of the polymer matrix. According to the peer-reviewed results discussed below, one could investigate the following theory to determine how the presence of MWCNTs accelerates the curing of each nanocomposite matrix: the physical adsorption between CNTs and epoxy resin increases the chemical reaction of resin and MEKP and accelerates the heat flow due to the good thermal conductivity of MWCNT crusts.

These assumptions are based on good shrinkage differences (see also Figure 2) concerning the effect of MWCNTs as a filler on the lowered gel time of curing systems. The development of the vigorous curing functions of an aliphatic type of thermosetting epoxy, as reported by Liu et al. [48], reinforced that gel formation has a good effect on the kinetics of the cure for the UPE/additive system. The affinity of the matrix in the curing system was found to have a strong influence on the curing of UPEs with additives. On the other hand, Chapartegui et al. [49] report a significant reduction in curing time due to the presence of MWCNTs accelerating the temperature-activated curing in comparison with pure epoxy resin. This is supported by the presence of MWCNTs, which initiates the early curing phase. At concentrations of 0.2–0.27 wt.% (see Figure 3 and Figure 4), the physical network between MWCNTs and the epoxy matrix slows down the dispersion-controlled process but accelerates the curing process with a lower shrinkage rate.

The decrease in gel time that was seen is thought to be caused by chemical reactions between the thermosetting epoxy matrix and the MWCNTs. This is based on results from the relevant literature that show how MWCNTs react chemically. Specifically, the gel time of all CO_2_-cured samples show a somehow narrow time period in the cross-linking reaction of UPE and VE nanocomposite matrices, compared to air-cured samples. Hu et al. [36] also noted that the enhanced kinetic constant and decreased activation energy point to the plasticization impact of CO_2_, which at a relatively low temperature facilitates chain movement, promotes epoxy resin curing, and increases ultimate conversion. As a substitute, we must consider that the excellent thermal conductivity and good adsorption tendency of MWCNTs with the resins would contribute to heat flow in dispersions and may trigger a faster cross-linking process between the resin and hardener [50]. Gojny et al. [51] have also reported on the subsequent link between thermal and electrical conductivity, where it is shown that the formation of a penetrated network of CNTs also has a particular influence on shrinkage. The differences in shrinkage between CO_2_- and air-cured MWCNTs are most likely due to the shorter distances between the MWCNTs, which simplifies crystal lattice conduction by reducing boundary scattering. However, the enhancement of thermal conductivity with the CNT concentration is modest compared with the air-cured specimens, probably due to the high thermal resistance at the polymer/nanotube interface [52].

## 11. Effect of MWCNTs on Air-Cured and CO_2_-Cured Gel Time and Exotherm

**Figure 5:**
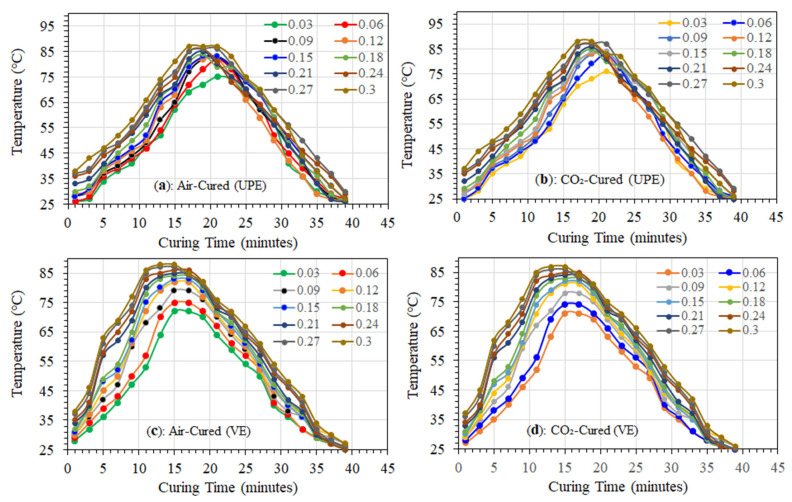
Rate of temperature increments during exotherm reaction as a function of various proportions of MWCNTs in UPE and VE nanocomposite matrix systems.

Figure 5a–d show how the temperature changed over time when the resin matrix was being cured at lower and higher quantities of MWCNTs. These time–temperature curves show the type of temperature change and the shortest amount of time needed for the resin to cure with the ideal resin-to-hardener ratio (16:1). Figure 5 shows that using lower concentrations of MWCNTs (0.03% to 0.18) reduced the air-cured cycle time linearly in both the UPE and VE nanocomposite matrix. Similar exotherm graphs were also observed in CO_2_-cured samples of MWCNTs, varying for each resin. However, in comparison to air-cured nanocomposite matrices, the CO_2_-cured gel time to hardness time cycle had a shorter cycle. This is most likely due to the non-existence of external scavengers such as O_2_, N_2_, humidity, and airborne particulate matter.

For different concentrations of MWCNT with UPE and VE matrices, we investigated the effect of curing temperature at fixed molar volumes of MEKP. However, we manually calculated the exothermic reactivity (gel to hardening) as a function of temperature vs. time [29]. Figure 5 also shows the duration of the gel transition as well as other curing phases, including the exotherm peak and peak exotherm time, the pace at which the temperature rises during the curing reaction, and the extent of cooling of both CO_2_- and air-cured specimens. The same figure makes it very evident that the lower the MWCNT content, the longer the gel time for the optimal resin to hardener ratio. This is mostly attributable to the increased integration of MWCNTs and their role as a connection between resins and precursors that interact with monomers to form smooth cross-links, particularly in CO_2_-cured samples in the absence of O_2_. Furthermore, the gel time variation appears to be greater (t ≈ 5–15 min) and the gel temperature lower in CO_2_-cured samples (see Figure 5b,d), and both thermoset nanocomposite matrices contain fewer MWCNTs. In addition, it is observed that the exothermic reaction peak temperature of the higher contents of MWCNT matrices in Figure 5b reach almost 89 °C with a shorter cross-linking time compared to air-cured (see Figure 5a) UPE samples. The higher temperature in the higher amount of MWCNT contents in the matrices probably revealed the shorter volume of resins. An almost similar behavior of exothermic reaction temperature were observed in Figure 5c,d, which could provoke a lower thermal gradient due to the higher amount of MWCNTs between the reinforced matrices’ surfaces and cores. However, the vinyl ester resin’s time laps are shorter (t ≈ 5–10 min) in gel time. This is probably due to the longer molecular chain, which serves to lessen the influence of residual stress due to the presence of MWCNTs. This is likely the reason why the gel duration in the VE matrices is longer than that in the UPE matrices [50]. The photographs in Figure 2 show good agreement, validating this explanation. In a recent review article of Cividanes et al. [53], it was reported that the curing process is significantly influenced by the level of MWCNTs in nanocomposite materials; a lower level of MWCNTs produced more uniformity in the resin matrix with medium viscosity. In their review article, it is reported that the epoxy resin cure is slowed down by heterogeneity and high viscosity, which lower the cure reaction heat.

However, the higher concentration of MWCNTs (0.2–0.3 wt%) in both nanocomposite matrices shows much shorter gel duration in both cured samples, which is about *t ≈ <* 7 min with a temperature at T ≈ 55–58 °C for UPE-8340 and *t* ≈ < 5 min at T = 62–65 ≈ for VE-922. It is suggested that a higher quantity of MWCNTs reduces the volume of resins, which could cause a shorter chain of resins. Moreover, unlike the lower quantity of MWCNTs, the higher quantity of MWCNTs increases the viscosity of resins. Consequently, the functional group of MEKP has a larger interaction with MWCNTs due to the OH group present in the hardener, which probably also contributes to the faster curing kinetic tendency in both curing techniques. Consequently, both nanocomposite matrices have a faster curing time. Moreover, compared to UPE-8340, VE-922 shows much lower gel time latencies and a faster exothermic process in both curing techniques.

## 12. Mechanical Properties

### Tensile Strength of UPE and VE Nanocomposites

As was explained in detail before [29], samples of lean epoxy matrices made of UPE/MEKP and VE/MEKPP, as well as their nanocomposites, were made by curing them in air and CO_2_. As described in the experimental section, the ASTM standard was used to test the tensile strength of each nanocomposite sample, even those with lean epoxy matrices. During the tensile strength tests, all of the samples worked well and shattered mostly in the middle of the testing area. This indicates that the MWCNT distribution within the two different epoxy matrices has a good structure and is probably homogeneous, and Figure 3 demonstrates good agreement with the visualized differences in various MWCNT contents in each nanocomposite sample. The measurements of the tensile strength of each sample were used to ascertain the most important properties, such as the maximum load (N), load at break (N), tensile strength (MPa), strain (%), and E-modulus (MPa).

**Figure 6:**
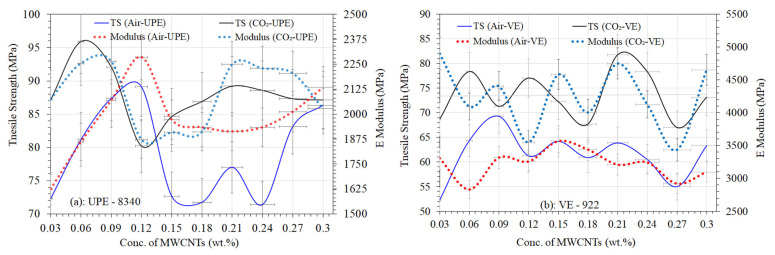
Tensile strength differences between air-cured and CO_2_-cured UPE-8340 and VE-922.

**Figure 7:**
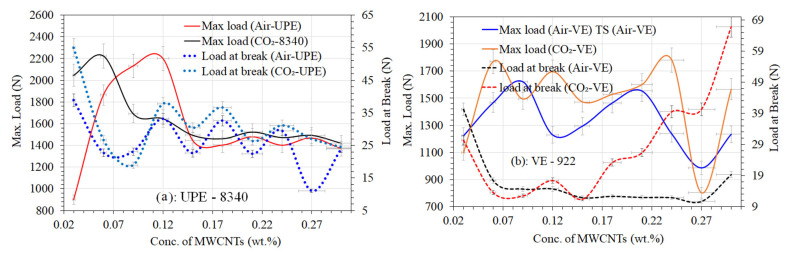
Maximum load differences between air-cured and CO_2_-cured UPE-8340 and VE-922.

Figure 6a and Figure 7a show how the different MWCNTs ratios affect the tensile strength, E-modulus, maximum load, and load at break of two different nanocomposite matrices cured in the air and CO_2_ chambers. The MWCNT contents in both nanocomposite matrices encourage noticeable improvements in tensile strength compared to those without the MWCNT matrices (see Figure 6a,b), and the differences with lean composite matrices can be readily seen in our earlier published work [29]. It was observed that both epoxy nanocomposites had almost 1.5 to 3 times the tensile strength of a lean epoxy matrix [29]. This is because MWCNTs act as a bridge in the nanocomposite matrices, which may help resins and hardeners make structures that are smooth. However, all of the CO_2_-cured nanocomposites showed much better mechanical properties, and we believed that this could be because of the absence of oxygen and humidity. Typically, the free O_2_ gas in the air can not only inhibit the cross-linking between resins and precursors but also reduce the tensile strength of epoxy composites.

From Figure 6a,b, it was noticed that the VE-922 matrix (cured under air and CO_2_), on the other hand, does behave very differently from UPE-8340 and has a relatively low tensile strength at different MWCNT contents. Despite the fact that the dispersion in the polymer matrix was achieved using high-intensity sonication, it could be most likely due to a lack of MWCNT interaction with the functional group of esters in the polymer of the VE matrix, particularly at higher MWCNT contents. Nonetheless, the air-cured UPE-8340 with 0.12 MWCNTs has a higher tensile strength of 89.12 MPa and an E-modulus ≈ 2285.9 MPa at a maximum load of ≈2201.3 N. The CO_2_-cured UPE nanocomposites, on the other hand, contain 0.09 MWCNTs and have a maximum tensile strength of ≈95.8 MPa and an E-modulus of ≈2255.04 MPa at a maximum load of ≈2222.9 N. However, the higher contents of MWCNTs in both nanocomposite matrices show lower tensile strengths and E-moduli at maximum load in both curing techniques. It is shown that during CO_2_ curing, the absence of moisture could reveal the good dispersion of a lower content of MWCNTs, but also perhaps significant adhesion between the resin matrix and MWCNTs. The similarity was also observed in CO_2_-cured VE nanocomposite resins. The air-cured VE-922 with 0.09 MWCNTs has a higher tensile strength of 69.32 MPa and an E-modulus ≈ 3319.44 MPa at a maximum load of ≈ 1621.8 N. The CO_2_-cured VE nanocomposites containing 0.06 MWCNTs have a maximum tensile strength of ≈ 78.34 MPa and an E-modulus of ≈ 4095.738 MPa at a maximum load of ≈ 1770.12 N.

The significant physisorption interaction between MWCNTs and the functional groups present in both polymer matrices, possibly the phthalic acid group of UPE, which shows efficient load transfer from the matrix to the MWCNTs, is primarily responsible for the enhancement of the tensile strength and E-modulus at maximum load with a low content of MWCNTs and led to an increase in the overall tensile properties of the nanocomposites. A number of studies [54,55,56] reported that functionalized MWCNTs can lead to the higher tensile properties of MWCNTs even though the higher content of MWCNTs shows a higher tensile strength tendency in nanocomposites. Similar findings were also reported by Hu et al., [57] who found that the impact strength and heat resistance of the epoxy matrix were significantly improved by the addition of MWCNTs modified via a surface carboxylation reaction. However, in this study, as nonfunctionalized MWCNT content in both epoxy nanocomposites increased, the composite’s tensile characteristics declined, and it is believed that the higher viscosity of both resins was unable to disperse the MWCNTs. This can be a result of the somewhat low MWCNT dispersion in the nanocomposite, brought on by an increase in MWCNT content. In addition, the higher the content of MWCNTs in both resins’ matrices, the larger the cavities that could be created during the fabrication process [55,56], which would have an impact on the nanocomposites’ overall tensile capabilities and reduce their composite features. Makki et al. [56] report that using the MWCNT contents evidently results in maximum force values that are greater than either free samples of MWCNTs or free samples of the epoxy resin matrix. This demonstrates how the maximum force of the test samples is impacted by MWCNTs.

The maximum load and load at break of both nanocomposite samples are shown in Figure 7a,b. The trends followed a similar pattern, as shown in Figure 6a,b. The maximum load of UPE-8340 and VE-922 improves with the addition of lower contents of MWCNTs. The air-cured UPE-8340 achieves its maximum load of ≈2201.29 N and its load at break is about ≈33.14 N with the addition of ≈0.12 wt.% of MWCNTs, and the minimum load at break is 10.9 wt.% of MWCNTs in the UPE-8340 nanocomposite matrix. The CO_2_-cured UPE-8340, on the other hand, has a nearly 1.1-fold higher maximum load and a ≈26.73 N load at break. The lower maximum load of 80.2 N and the load at break of about 37.8 N of CO_2_-cured UPE-8340 were achieved along with the highest maximum load of about 69.3 N and the maximum load at break of ≈14.69 N of air-cured VE-922 nanocomposite containing 0.09 wt.% of MWCNTs. The air-cured VE-922 sample, which contains 0.03 weight percent of MWCNTs, achieved the lowest maximum load and a load at break of about 52.25 and 40.33 N, respectively. The CO_2_-cured VE-922 shows significant differences in maximum load and load at break compared to the air-cured VE-922 nanocomposite samples, as can be clearly observed in Figure 7b.

There are a number of reasons why lower concentrations of non-functionalized MWCNTs improve the tensile performance of both air-cured and CO_2_-cured nanocomposite matrices. The first interpretation is that the nanocomposite matrix is held in place by the MWCNTs, which serve as a bridge moiety in the structure. The same thing was seen in Hu’s [57] studies, which showed that adding lower concentrations of MWCNTs to the epoxy matrix made it much more stable mechanically and thermally. The improved the high aspect ratio (length/diameter) of the MWCNTs and made it possible to move a load to the carbon nanotubes [57]. Another step in the process of creating MWCNTs/polymer nanocomposites is interfacial adhesion between the UPE-8340 and VE-922 nanocomposite matrix and the MWCNTs.

## 13. Interfacial Shear Stress

The non-functionalized MWCNTs with lower contents enhance the performance of the nanocomposite materials as a whole due to their easy dispersibility and strong interfacial physisorption compatibility with the thermoplastic matrix. MWCNTs surfaces, which influence both the wettability and dispersibility of the liquid precursors as well as the strong attraction interactions between the MWCNTs themselves, may benefit greatly from the functional groups of UPE and VE. These improve the interfacial bonding of the composite [57,58,59].

**Figure 8:**
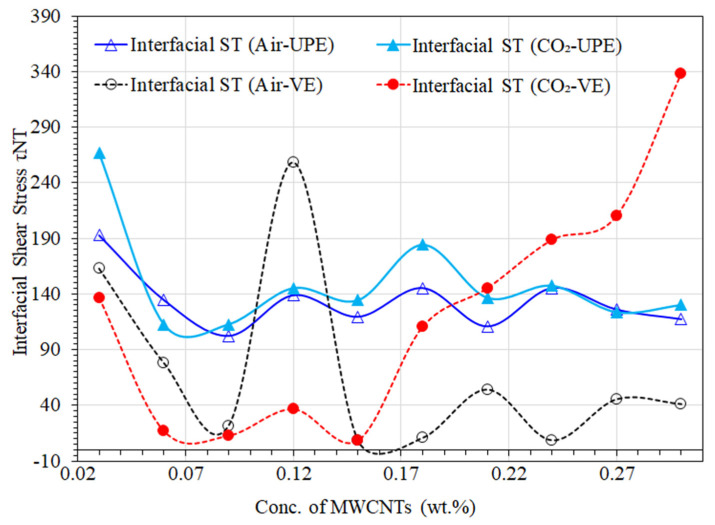
Interfacial shear stress of air- and CO_2_-cured UPE and VE matrices with various quantities of MWCNTs.

Figure 8 shows the interfacial shear stress of air- and CO_2_-cured UPE and VE matrices with various quantities of MWCNTs. It has been found that the strength of UPE-8340 and VE-922 nanocomposites is directly influenced by the quantity of nonfunctionalized MWCNTs in the matrix. As seen in Figure 6 and Figure 7, the higher the content of MWCNTs, the lower the tensile properties. As can be seen in Figure 8, the higher contents of MWCNTs in the polymer matrix show higher interfacial shear stress except for 0.03 wt.% of MWCNTs. The lowest MWCNT contents are likely to be 0.03–0.06 wt.%, indicating an appropriate dispersion with the polymer matrix and thus an ability to sustain the large loading stress in the interphase region and high interfacial shear strength; this tendency is expected. A low interfacial shear strength, however, is unable to resist significant stress and splits or shatters when it faces higher mechanical and thermal loads. On the other hand, the higher content of MWNTs in the polymer matrix shows higher shear stress. This is attributed to the nanocomposite’s tight structural morphology in both polymer matrices. The capacity of the interphase area for stress transfer between the polymer matrix and the MWCNTs can be said to be determined by the interfacial shear strength. As a result, Equations (5) and (6) express how the interfacial shear strength directly influences the tensile strength of nanocomposites [60].

The increased MWCNT content (0.27–0.3 wt.%) in both nanocomposite matrices revealed a thick structural interphase, which is subject to voidless interaction between the polymer matrix and the MWCNTs. This is indicative of strong interfacial adhesion. In this instance (higher content), the MWCNTs easily distribute the stress from the polymer matrix, which demonstrates that they reinforce the polymer matrix. A thin interphase, on the other hand, denotes inadequate interfacial adhesion between the polymer and the MWCNTs, which makes the nanocomposites fail to withstand the loading tension and liftoff from the sample. As a result, a thick interphase strengthens nanocomposites, and the developed sample effectively illustrates how interphase thickness affects nanocomposite strength. Other articles [38,60,61] supported these findings by describing the same effect of interphase size on the strength and modulus of nanocomposites.

## 14. Flexural Strength

The effects of various quantities of MWCNTs on tensile strength, E-modulus, and load at break are covered in the above sections. It was found that with the lower contents (0.03–0.2 wt.%) of MWCNTs in both nanocomposite matrices, the tensile strength and E-modulus are increased; however, the load at break is decreased. Moser and Feuchter [62] found that the mechanical properties of the nanocomposite matrix were improved when filler or nonfunctionalized CNTs were added to thermoset resins at a level of less than 1 wt.%.

**Figure 9:**
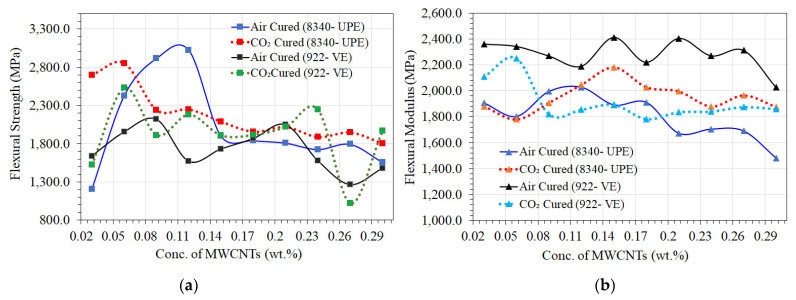
Flexural strength and modulus of UPE and VE nanocomposite samples. (**a**) flexural strength comparison of air-, CO_2_-cured UPE and VE nanocomposite; (**b**) flexural modulus comparison of air-, CO_2_-cured UPE and VE nanocomposites.

Among the tensile strengths, the flexural strengths and flexural modulus are two of the most typical types of loads found in desalination applications, particularly pressure-driven membrane housing and water transmission lines. As a result, they are crucial for determining the flexural strength characteristics of polymers and composite materials [19]. Each nanocomposite sample accordingly underwent a flexural strength test in accordance with ASTM D 790 guidelines. The flexural modulus (Figure 9b) was calculated from the slope of the stress–strain curve. The purpose of this test is normally to estimate the maximum stress and strain that an external or internal load may create. Figure 9a,b show how the flexural strength and modulus of nanocomposite samples are affected by different MWCNT contents. From Figure 9a, the lower contents of MWCNTs show the higher flexural strength of the nanocomposite sample in both curing techniques. It was found that lower MWCNT contents produced minor flexibility, most likely due to the smooth cross-linking density of the nanocomposite samples. Technically, it is a good idea to use composite materials such as these in desalination, especially for water transmission lines.

There may have been more cross-links in the nanocomposites with higher MWCNT contents (more than 0.25 wt% of MWCNTs), and the samples were more brittle.

Furthermore, the mechanical properties of all nanocomposite samples improve when lower contents of nonfunctionalized MWCNTs are added to UPE and VE resins. Compared to VE, the UPE nanocomposite’s flexural strength is almost twice (0.12 wt.% of MWCNTs) as high, but it shows a lower flexural modulus in air-cured samples, and the sample suggests that perhaps the CO_2_-cured nanocomposite samples show optimized mechanical properties compared to air-cured samples. It is revealed that the lower content of MWCNTs may restrict the maneuverability of polymer cross-link chains during the curing reaction and modify the final structure of the cross-linked network of the UPE and VE. In addition, firming of intermolecular strengths between the thermoset resins and MWCNTs may occur, which facilitates an active transfer of internal stresses between both the thermoset polymer and MWCNTs, leading to the enhancement of the flexural properties of UPE/MWCNTs and VE/MWCNTs nanocomposites.

## 15. Surface Characterizations

Thermoset epoxies, in particular UPE and VE types of polymers, are well known for their exceptional barrier qualities in a variety of environmental circumstances. In order to optimize their structural and surface properties for water application, a number of additives are added to thermoset polymers to optimize their hydrophobic and hydrophilic stability [63]. A number of macroscopic physical properties, such as moisture adsorption, cross-linking, and degradation of reinforced composites, were studied with the addition of additives. Moreover, an essential aspect of thermoset-reinforced matrices that determines their stability in practical applications is the equality or optimization between hydrophobic and hydrophilic properties [63]. Comprehensive comparisons were carried out to elucidate the sorption behavior of two different kinds of water, DIW and RO brine, on the surfaces of air-cured vs. CO_2_-cured UPE-8340- and VE-922-reinforced composites. Specifically, the change in the hydrophilic/hydrophobic balance in the presence of various contents of MWCNTs was discussed. Contact angle (CA), degree of water adsorption (DA), and surface free energy (SFE) measurements in these nanocomposites gave an indication of a change in surface properties with respect to the lower to upper contents of MWCNTs in both reinforced nanocomposites. 

**Figure 10:**
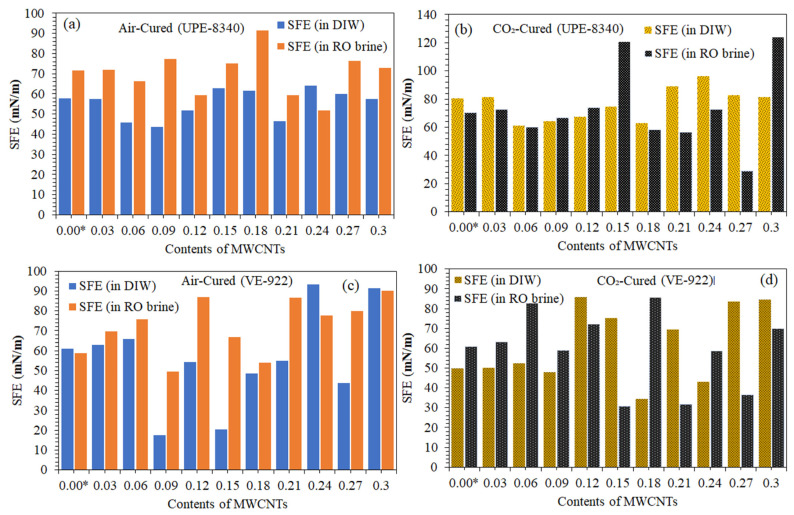
Surface energy of various amount of MWCNT nanocomposites in DIW and seawater reverse osmosis brine water (79,000 ppm). (**a**) SFE of air-cured UPE nanocomposites in DIW and RO brine; (**b**) SFE of CO_2_-cured UPE nanocomposites in DIW and RO brine; (**c**) SFE of air-cured VE nanocomposites in DIW and RO brine; (**d**) SFE of CO_2_-cured VE nanocomposites in DIW and RO brine.

Table 3 and Table 4 and Figure 10a–d summarize the comparison measurements of de-ionized water (DIW) and the RO brine CA, DA, and SFE of air-cured vs. CO_2_-cured UPE-8340- and VE-922-reinforced lean epoxy composites and nanocomposites. Table 3 shows the CA and DIW of air-cured UPE-8340 lean reinforced composite (std.) is 77.22°, which is a 1.344° higher CA than the CO_2_-cured std. Both curing techniques for std. composites exhibit poor hydrophilic properties. However, after adding the MWCNTs to the UPE matrix, the CA turned partially hydrophobic. The CO_2_-cured UPE nanocomposites had 1–3 times higher CA than the air-cured UPE nanocomposite. Similar trends were also observed in the degree of DIW adsorption, which shows that the lower the CA gain, the higher the degree of DIW adsorption. In addition, the SFE shows a similar pattern (see Figure 10a,b).

Additionally, the surface free energies were calculated from the contact angle data. As expected, with the addition of MWCNTs in air and CO_2_-cured UPE nanocomposites, the contact angle increased with a decrease in surface free energy (see Figure 10a,b). Despite this, the surface became hydrophobic due to the hydrophilic nature of the phthalic anhydrate group in UPE, as well as their presence on the surface of the samples. In this system, the most hydrophobic sample was the neat epoxy sample, while the blend containing lower contents of MWCNTs in the UPE nanocomposite showed comparatively higher hydrophobicity. It is interpreted that the lower contents of MWCNTs in UPE align the cross-linking chain and reduce the polarity of phthalic anhydride due to the strong physisorption interlocking with the phthalic group of UPE.

Nevertheless, the lower content of MWCNTs in UPE nanocomposites revealed higher surface contact properties compared to higher contents (0.2–0.3 wt.% of MWCNTs). Unlike DIW surface contact properties, RO brine water revealed optimized tendencies and stability in salt concentrations for UPE-8340 (Table 3), which shows considerable potential for the desalination sector. Similar trends were also seen in Figure 10b, where the SFE of all CO_2_-cured samples shows significant stability in concentrated salt brine water. On the other hand, the VE-922 nanocomposite-reinforced samples show a noticeable higher CA compared to std. (lean epoxy) and, lower DA, and lower SFE (see Figure 10c,d) compared to std. VE-922 composite samples (Table 4). However, all CO_2_-cured reinforced composites revealed optimized and significant properties.

Two different water behaviors (DIW and RO brine) were studied, and they showed similar trends in both reinforced composite systems. The equilibrium water uptake by both cured nanocomposites was found to be strong for RO brine. Additionally, the poor hydrophilicity and partial hydrophobicity of both nanocomposite samples could somewhat be interpreted as a balanced hydrophobic/hydrophilic structure due to the addition of a lower content of MWCNTs with a sustainable dispersion in the resins, which was also responsible for the anomalous behavior for both waters. The fractional free volume was found to decrease with an increasing MWCNT content, and the drop in free volume was related to a specific, probably symmetric structure and hence decreased the water uptake (Table 3 and Table 4 and Figure 10a–d). Hence, the air-cured nanocomposite sample shows moderate surface contact with DIW and RO brine, probably due to the presence of humidity and moisture in the reinforced dope. Thus, the water molecules in the epoxy blends are primarily located within the epoxy phase, and water uptake is dependent on the total epoxy content and structure of nanocomposites regardless of the MWCNT contents used in this study; however, the air-cured UPE and VE nanocomposites demonstrated good RO brine resistance despite the higher MWCNT contents in the component.

Additionally, with fewer MWCNTs present, the overall SFE of both air-cured nanocomposites dropped from 15 to 2 mN/m. In contrast to the air-cured reinforced composites, the CO_2_-cured nanocomposites (UPE and VE) showed some improvement in SFR (see in Figure 10b,d). The MWCNTs may have enhanced each composite’s surface tendencies, according to certain theories. The results showed that all CO_2_-cured nanocomposite samples had more pronounced hydrophobicity as a result of sorting the polar groups of the epoxy, which resulted in a substantial rise in hydrophobicity.

Due to the lower MWCNT content and its interaction with the monomers, the higher contact angle in both reinforced polymeric matrix curing procedures likely exhibited lesser oxidative degradation. Yasuda et al. [64] reported that the oxygen most likely causes this smooth hydrophobic layer to form at the interface during the curing process.

## 16. Conclusions

In this study, inadequate shrinkage curing procedures for UPE and VE with MWCNTs nanocomposite reinforcement were developed, and comparisons with standard (std.) reinforced matrices were established. The UPE and VE with the MWCNTs nanocomposite produced a thick interphase when cured in contact with a 3D-printed ABS dumbbell-shaped mold using a closed-chamber curing method with air and CO_2_. The ASTM D638-03 standard made it possible to repeat the warping of a dumbbell-shaped specimen in a closed room in a systematic way, which showed that the shrinkage was carried out on purpose (cure shrinkage plus thermal shrinkage). After curing, the relative molar volume of MWCNTs in VE and UPE resins changed from the interface to the bulk, making this interfacial layer thinner. The effects of air and CO_2_ curing on resin gel time and exotherms demonstrated that higher MWCNT contents have less impact on chemical and physical stress on the polymer matrix during the curing process.

The findings showed that the volume shrinkage for the MWCNT component was larger with a shorter gel duration, and it can be particularly clearly observed in UPE-8340 nanocomposite matrices. This phenomenon has traditionally been linked to an increase in the breakdown of the precursor’s acetone peroxide group, a highly oxidizing reagent, and the production of extremely reactive free radicals that interact with monomers to form cross-linkages. Compared to air curing, CO_2_ curing has been shown to make specimens with less shrinkage, better tensile and flexural strength, and better contact with water. It is shown that the flexural modulus of the resin rises from the interface to a certain depth. This could have a large effect on how well UPE and VE nanocomposite matrices with lower initiator volume ratios work under mechanical stress.

## Figures and Tables

**Table 1:** The composition of UPE/MWCNT and VE/MWCNT reinforced nanocomposite.

Samples	MWCNTs	VE (Hetron 922)	UPE (Siropol 8340)	MEKP
	gm	mL		
1	0.03	20	20	1.2
2	0.06			1.2
3	0.09			1.2
4	0.12			1.2
5	0.15			1.2
6	0.18			1.2
7	0.21			1.2
8	0.24			1.2
9	0.27			1.2
10	0.3			1.2

**Table 2:** Dimensions of MWCNTs.

Dimensions of MWCNTs		Average Dimensions	Average Dimensions in Meter
Outside Diameter (*D*)	8–18 nm	13 nm	1.3 × 10^−9^
Inside Diameter (*d*)	5–10 nm	7.5 nm	7.5 × 10^−9^
Length (*L_c_*)	1 × 10^4^–3 × 10^4^ nm	2 × 10^4^ nm	2.0 × 10^−9^

**Table 3:** (a) Comparison of DIW contact behavior on the surfaces of air-cured vs. CO_2_-cured UPE-8340-reinforced composites. (b) Comparison of seawater brine contact behavior on the surfaces of air-cured vs. CO_2_-cured UPE-8340-reinforced composites.

Contents of MWCNTs	Surface Behavior in Deionized Water (DIW)			
	Air-Cured (UPE-8340)		CO_2_-Cured (UPE-8340)	
	CA (θ)	DA	CA (θ)	DA
(a)				
0.00 *	77.220	0.010	75.876	0.006
0.03	83.85	0.008	82.680	0.004
0.06	84.47	0.007	84.490	0.004
0.09	84.48	0.007	84.750	0.004
0.12	80.553	0.008	81.860	0.005
0.15	81.760	0.006	84.24	0.004
0.18	83.310	0.008	85.05	0.006
0.21	84.750	0.009	86.680	0.007
0.24	85.203	0.006	88.660	0.004
0.27	84.170	0.008	87.56	0.006
0.3	82.612	0.005	85.010	0.004
(b)				
0.00 *	79.320	0.009	77.634	0.004
0.03	90.152	0.005	91.870	0.002
0.06	91.72	0.006	92.749	0.003
0.09	92.68	0.005	93.195	0.004
0.12	93.753	0.004	95.740	0.001
0.15	95.160	0.004	95.378	0.002
0.18	95.810	0.005	96.701	0.004
0.21	95.950	0.005	95.974	0.004
0.24	97.423	0.004	98.436	0.003
0.27	98.031	0.004	98.972	0.003
0.3	96.412	0.004	97.017	0.004

Contact angle (CA); degree of water adsorption (DA); and * shows the standard (Std) of the lean epoxy matrices, i.e., five parts (mL) UPE and VE and 0.3 parts(mL) hardener (methyl ethyl ketone peroxides, or MEKP).

**Table 4:** (a) Comparison of DIW contact behavior on the surfaces of air-cured vs. CO_2_-cured VE-922-reinforced composites. (b) Comparison of seawater brine intake behavior on the surfaces of air-cured vs. CO_2_-cured VE-922-reinforced composites.

Contents of MWCNTs	Surface Behavior in Deionized Water (DIW)			
	Air-Cured (VE-922)		CO_2_-Cured (VE-922)	
	CA (θ)	DA	CA (θ)	DA
(a)				
0.00 *	79.913	0.010	81.230	0.006
0.03	80.950	0.002	84.940	0.001
0.06	81.290	0.002	89.350	0.002
0.09	81.940	0.004	90.180	0.002
0.12	84.000	0.002	85.560	0.002
0.15	80.900	0.001	85.510	0.002
0.18	91.560	0.001	93.810	0.002
0.21	93.510	0.004	91.150	0.001
0.24	94.810	0.003	97.170	0.001
0.27	93.010	0.001	104.080	0.001
0.3	95.20	0.002	105.860	0.001
(b)				
0.00 *	78.725	0.006	80.525	0.005
0.03	85.85	0.003	87.550	0.002
0.06	91.800	0.001	93.760	0.003
0.09	91.99	0.003	95.790	0.002
0.12	93.639	0.001	96.939	0.003
0.15	95.290	0.003	98.890	0.002
0.18	97.162	0.002	100.262	0.002
0.21	97.774	0.004	99.574	0.002
0.24	95.617	0.003	97.717	0.003
0.27	95.010	0.002	97.210	0.002
0.3	92.208	0.003	95.709	0.003

Contact angle (CA); degree of water adsorption (DA); and * shows the standard (Std) of the lean epoxy matrices, i.e., five parts (mL) UPE and VE and 0.3 parts(mL) hardener (methyl ethyl ketone peroxides, or MEKP).

## Data Availability

All the research work and data collection were produced at the Center of Excellence in Desalination Technology, King Abdulaziz University, Jeddah.
